# Generative Constraint: How Baroque Musical Structures Scaffold Creativity in Neurodiverse Learners

**DOI:** 10.3390/jintelligence14090217

**Published:** 2026-09-09

**Authors:** Seçil Soytok Nalçacı, Kutup Ata Tuncer

**Affiliations:** Department of Music Education, Manisa Celal Bayar University, 45900 Manisa, Turkey

**Keywords:** creativity, special education, neurodiversity, Baroque music, partimento, generative constraint, mini-c, music education, emotional regulation, conceptual framework

## Abstract

Creativity is a central educational aim, yet learners in special education are too often left out of discussions of how to cultivate it. Music is frequently proposed to support them, and Baroque music especially, but the usual rationale, that such music directly improves cognition, rests on the discredited Mozart-effect literature. This paper offers a different foundation. It advances the thesis of generative constraint: structured musical environments do not enhance cognition but establish the affective and attentional conditions, principally through regulation, under which everyday (mini-c) creativity can emerge in neurodiverse learners. Baroque architecture, including the ground bass, theme and variation, and the historical pedagogy of partimento, is a paradigmatic instance. What sets it apart from a mere repeating background is its functional-harmonic directionality: unlike a static ostinato, a Baroque ground carries an implied motion of tension and resolution, and it is this directional pull, not repetition as such, that a learner’s choices engage, and that offers a controlled means of working against rigidity rather than reinforcing sameness. Because neurodiverse learners differ, the model treats sensory and arousal profiles as moderators, specifies when the approach may fail or do harm, and measures creative variation to separate it from stereotyped behaviour. The contribution is conceptual: a testable model and a single-case research agenda.

## 1. Introduction

Creativity occupies a prominent place among the competencies that contemporary education seeks to develop, and the field has increasingly treated it as a definable and researchable construct rather than an ineffable gift (Plucker et al., 2004; Runco & Jaeger, 2012). That progress has not been evenly shared. Discussions of how to cultivate creativity have centred on typically developing learners, while students in special education, including autistic students and those with attention-related or learning differences, appear far less often, despite having as much claim to creative development as any other group (Elisondo, 2025). Addressing this imbalance is partly a matter of equity and partly a matter of taking seriously the creative capacities that a strength-based view of neurodiversity would lead one to expect.

To make the proposal concrete from the outset, a representative activity can be described in a sentence. A learner sits at a keyboard or with simple software while a short two-bar bass pattern repeats, supplied by the teacher, an app, or a recording; the learner’s task is only to shape a melody, or even a few chosen notes, above that unchanging foundation. Everything below is fixed, predictable, and safe; the small, bounded space above is where the learner’s own choices are made. The remainder of this paper explains why an activity of this kind, and a graded family of others like it, may support creativity in neurodiverse learners, and it does so without any appeal to the idea that the music itself sharpens the mind.

Music is among the most commonly proposed vehicles for supporting these learners. The rationale usually offered, however, is weak. It holds that the mathematical order and steady tempo of classical and Baroque music directly sharpen attention, memory, or intelligence, an idea descended from the Mozart-effect research of the 1990s. That literature has not survived scrutiny: the largest meta-analysis finds no meaningful cognitive benefit specific to the music (Pietschnig et al., 2010), and the apparent effects are better explained by transient arousal and mood (Thompson et al., 2001). Claims of this kind are now recognised as educational neuromyths, loosely tied to genuine science but unsupported by it (Dekker et al., 2012). A proposal for using Baroque music in special education that rests on this foundation inherits its weakness.

This paper retains the practical intuition that Baroque music can support creativity in these learners but replaces its justification. The central claim, developed as the thesis of generative constraint, is that structured musical environments do not improve cognition; they establish the conditions, above all the regulation of arousal and affect, under which creative behaviour can occur. The relevant creativity is not eminent achievement but the everyday, learning-embedded creativity that Kaufman and Beghetto (2009) call mini-c, and the relevant evidence is the cautious body of work showing that music-based intervention in autism supports regulation and social-emotional engagement rather than broad cognitive gain (Geretsegger et al., 2022).

Because “neurodiverse learners” is not a homogeneous category, the argument is framed throughout as conditional rather than universal. Autistic learners and learners with attention-related differences can have markedly different, sometimes opposite, sensory and arousal needs, and the model that follows is built to accommodate that variation rather than to paper over it; the point is developed in Section 3.2 and formalised as a set of moderators in Section 6. A framework that promises the same benefit to every learner regardless of profile would, for that very reason, be less scientific, not more.

The reframing gains its force from a feature of the music itself. Well-chosen constraint is not an obstacle to creativity but an engine (Stokes, 2006), and Baroque musical architecture is a particularly clear embodiment of productive constraint. A ground bass fixes a foundation while inviting free variation above it; theme-and-variation form prescribes a bounded set of transformations; and the historical practice of partimento and figured bass was, in its own time, a complete pedagogy for cultivating creative fluency by means of structure (Gjerdingen, 2007; Sanguinetti, 2012). The structure is therefore not imposed on the music from the outside but is the music’s own, and a central Baroque pedagogy was already a method for teaching structured improvisation.

The contribution of this paper is conceptual rather than empirical. It does not report data; it specifies a model precise enough to be tested and indicates how a test would proceed. Three questions organise the argument: how the generative-constraint framework explains the way structured musical environments support creativity in neurodiverse learners; how Baroque musical forms instantiate that framework; and which factors moderate the strength of the effect, and how its outcomes might be recognised and measured. The paper first fixes the conceptions of creativity on which it relies and identifies mini-c as the appropriate scale, then develops the generative-constraint thesis and shows in detail how Baroque forms embody it. From that analysis, it builds a single model and its translation into classroom practice, specifies the learner, teacher, classroom, and institutional factors that moderate the effect, and turns to the outcomes the approach should and should not be expected to produce and how they might be measured. It closes by deriving testable predictions, identifying the single-case designs suited to evaluating them, and setting out the conditions under which the model would be disconfirmed.

## 2. Conceptualizing Creativity

The argument of this paper draws on a small number of well-established ideas in creativity research, developed over a literature that runs from the mid-twentieth century to the present. This section sets them out, both to fix terminology and to identify, at each step, the version of the idea that is appropriate for special education. Three commitments organise what follows: a working definition of creativity, a model that locates the relevant scale of creativity for this population, and a componential view that gives the environment a constitutive role.

### 2.1. Defining Creativity

Contemporary creativity research converges on a definition with two requirements: a creative product is one that is both novel and appropriate, useful, or fitting within a given context (Plucker et al., 2004). Novelty alone is insufficient, since a response that is merely unusual may be random or inapt. Appropriateness supplies the second criterion and, with it, a constraint internal to the very definition of creativity. Plucker and colleagues argue that the field’s slow progress owed much to imprecision on exactly this point, and that careful definition is a precondition for cumulative work. This two-part criterion is now the field’s most widely shared reference point; Runco and Jaeger (2012) call novelty paired with usefulness the standard definition of creativity, and it continues to anchor recent syntheses of the field.

One clarification is essential for the special-education setting, and it concerns who decides what counts as appropriate. If appropriateness is read as conformity to mainstream, neurotypical expectations of what a “good” product should look like, the criterion risks becoming an instrument for disciplining precisely the divergent expression a strength-based approach means to support. The reading adopted here is different and narrower: appropriateness is fit to the task’s own constraints and to the learner’s own intention, judged where possible with the learner rather than for them. A variation is appropriate if it answers the bounded problem the activity poses, not if it satisfies an external aesthetic norm. Understood this way, appropriateness remains a genuine constraint, one that distinguishes creativity from randomness, without smuggling in a demand for neurotypical conformity. The point is taken up again in the treatment of measurement in Section 7.2, where originality is assessed against the learner’s own repertoire rather than a population norm.

The modern study of creativity is usually dated to Guilford’s (1950) presidential address to the American Psychological Association, which criticised the scientific neglect of the topic and proposed a research programme built on the factor-analytic study of intellectual abilities (Guilford, 1950). Central to that programme was the notion of divergent production, later widely known as divergent thinking: the capacity to generate many varied responses to an open-ended prompt, as distinct from the convergent thinking that seeks a single correct answer (Guilford, 1967). Torrance operationalised this line of work in a battery of tasks scored for fluency, flexibility, originality, and elaboration (Torrance, 1966), dimensions that remain in wide use more than half a century later. For present purposes, divergent thinking is treated as one important component of creative ability rather than as its whole; the definition above, which requires appropriateness as well as novelty, guards against equating creativity with sheer ideational output.

### 2.2. The Four C Model and the Appropriate Scale

Defining creativity does not settle the question of which creativity is at issue in a special-education classroom. The Four C model offers the necessary distinction (Kaufman & Beghetto, 2009). It separates Big-C, the eminent creativity of acknowledged historical contribution, from Pro-c, the competence of accomplished professionals, and both from the little-c of everyday creative activity. To these, the model adds a fourth and, for present purposes, decisive category: mini-c, the novel and personally meaningful interpretation that is intrinsic to the process of learning itself (Beghetto & Kaufman, 2007).

Mini-c is the appropriate target for the approach developed here. The aim of a generative-constraint activity is not to produce eminent works, nor even, in the first instance, the shareable products of little-c, but to support the learner’s own meaningful construction of musical ideas in the course of learning. Setting the expectation at the level of mini-c is not a lowering of ambition; it is a correct alignment between the construct and the setting. It also makes the outcomes of such work assessable on their own terms, a point developed in Section 7. The distinction has entered large-scale practice as well: the OECD’s PISA 2022 assessment of creative thinking was framed explicitly around little-c, the everyday creativity that can be developed through education, rather than around eminent achievement (OECD, 2024).

### 2.3. The Componential View: Creativity and Its Environment

The third commitment concerns the place of the environment. Amabile’s componential theory holds that creative performance draws on three intra-individual components, namely domain-relevant skills, creativity-relevant processes, and task motivation, together with the surrounding social and physical environment, which the theory treats as a genuine component rather than as a mere context (Amabile, 1983, 1996). On this view, intrinsic motivation is particularly conducive to creativity, and the conditions that support or undermine it are part of the creative mechanism.

This matters for the present argument in two ways. First, it licenses the claim, central to Section 5, that designing the environment is a way of acting on creativity itself rather than merely arranging the circumstances in which it might happen to occur. Second, it connects creativity to the affective and motivational states that Section 3 places at the heart of the model. If the environment is a component of creative work, then a structured, secure, and appropriately motivating setting is not preparatory to the creative act but partly constitutive of it.

### 2.4. Creative Self-Beliefs and Creativity Anxiety

A fourth body of work concerns the beliefs and affective states that govern whether creative capacity is exercised at all. Two constructs are central to the present argument. The first is creative self-efficacy, the belief that one can produce creative outcomes (Beghetto, 2006); within the broader model of creative behaviour as agentic action, such creative self-beliefs function as a motivational engine that converts creative potential into creative action (Karwowski & Beghetto, 2019). Related work on creative mindsets shows that believing creative ability can grow, rather than being fixed, is positively associated with creative self-efficacy and with a willingness to engage (Karwowski, 2014). The second construct is creativity anxiety, an anxiety specific to creative thinking and distinct from general anxiety (Daker et al., 2020). It predicts poorer creative performance and, much like other domain-specific anxieties, encourages avoidance of the very situations in which creative skill might otherwise develop (Daker et al., 2023). In combination, these findings locate an important part of the conditions for creativity in the learner’s affective and motivational state: creative engagement is more likely where self-efficacy is high and creativity anxiety is low.

These commitments, namely a definition built on appropriateness, mini-c as the relevant scale, the environment as a constitutive component, and the creative self-beliefs and anxieties that gate creative action, frame the thesis that follows. They also point toward the population at its centre. Creativity in special education is best approached from a strength-based stance, one that asks what conditions allow a neurodiverse learner’s creative capacity to be expressed, rather than treating difference as a deficit to be remediated. Section 3 develops that stance into the generative-constraint thesis.

## 3. The Generative-Constraint Thesis

The central claim of this paper is that structured musical environments do not foster creativity by improving cognition. They foster it by establishing the affective and attentional conditions under which creative behaviour can occur. This distinction matters because the most common rationale for using “classical” music in educational and therapeutic settings rests on a different and far weaker premise: that the mathematical order and steady tempo of such music directly sharpen attention, memory, or intelligence. As Section 2 began to suggest, and as the evidence reviewed below confirms, that premise does not survive scrutiny (Pietschnig et al., 2010; Thompson et al., 2001). The position developed here replaces it with a mechanism that the evidence does support, drawing on the psychology of constraint (Stokes, 2006) and the componential role of the environment (Amabile, 1996), and that also happens to be a better fit for the strengths and needs of neurodiverse learners.

The argument proceeds in three steps. First, the right kind of constraint enables creativity rather than suppressing it. Second, structured and predictable environments are of particular value to neurodiverse learners, for reasons grounded in regulation rather than remediation, though the value is conditional on the learner’s profile rather than uniform. Third, when these two ideas are combined, music’s contribution can be restated cleanly: not a cognitive boost, but a regulated and bounded space in which everyday, learning-embedded creativity becomes possible.

### 3.1. Constraints as the Engine of Creativity

A persistent folk model treats creativity as the product of unbounded freedom, as though removing limits were the surest route to original work. The psychological literature points in the opposite direction. Stokes (2006), in her account of breakthrough across the arts and sciences, argues that it is precisely the self-imposed, well-considered constraint that drives creative advance. Her examples are instructive for the present case because several are musical: a composer who restricts available harmonic or rhythmic material is not impoverished by the restriction but is pushed toward solutions that would not otherwise have been found. Constraint, on this view, does not narrow the creative space arbitrarily; it structures the search through it, converting an unmanageably open problem into a tractable one.

This is consistent with how creativity has come to be defined in the field. The standard definition treats a creative product as one that is both novel and appropriate to its context (Plucker et al., 2004); appropriateness is itself a constraint, since unconstrained novelty is merely random. It is also consistent with Amabile’s componential account, in which the social and physical environment is not a backdrop to creative work but a constitutive component of it (Amabile, 1983, 1996). A good environment, in other words, is part of the creative mechanism rather than a precondition external to it. The pedagogical implication is direct: the task of the educator is not to remove structure in the hope that creativity will rush into the vacuum, but to design the constraints that make a productive search possible.

### 3.2. Why Structure Especially Serves Neurodiverse Learners

If well-chosen constraints benefit creative work in general, it is of heightened value for many neurodiverse learners, including autistic students and those with attention-related or learning differences. The reasoning here is deliberately strength-based rather than deficit-based, in keeping with the neurodiversity paradigm’s argument that autistic ways of perceiving and attending are differences, and at times strengths, rather than deficiencies to be corrected (Mottron et al., 2006; Pellicano & den Houting, 2022). Predictable structure is not framed as a crutch that compensates for what a learner lacks; it is framed as an environmental feature that reduces extraneous cognitive load (Sweller, 1988) and so frees attention and affect for the generative task at hand. This matters because meta-analytic evidence indicates broad, if variable, executive-function differences in autism (Demetriou et al., 2018), and open-ended, weakly structured activities place heavier demands on exactly these functions than clearly bounded ones do. A bounded task lowers those demands and makes sustained engagement more attainable. In this respect the structure does what scaffolding was originally meant to do: in Wood et al.’s (1976) account, an adult controls the elements of a task that lie beyond the learner’s reach so the learner can concentrate on those within it, and one of their named scaffolding functions is precisely the reduction in degrees of freedom, which is precisely the constraint this paper attributes to musical form (see also Vygotsky, 1978).

It is essential, however, to distinguish reducing extraneous load from reducing the intrinsic difficulty of the material, because the two come apart in a way that matters for the more advanced Baroque practices discussed in Section 4. Structure of the kind proposed here lowers extraneous load, the load imposed by a task’s presentation and openness, by fixing a predictable frame and narrowing the space of choices; it does not, and is not meant to, abolish the intrinsic complexity of a demanding musical operation. The practical consequence is that the framework is graded. Its entry points, a repeating two-bar bass or a single-parameter variation, carry very low intrinsic load and are appropriate for most learners; the full historical apparatus of figured-bass realisation carries high intrinsic load and is emphatically not proposed as a starting point, or as a universal one. Where an activity’s intrinsic demand exceeds a given learner’s current capacity, the response is to simplify the constraint, not to abandon the principle. Load, in short, is titrated to the learner, and the claim that structure reduces extraneous load is fully compatible with the recognition that a real musical task must still be pitched at a reachable level of intrinsic difficulty.

Predictability also carries an affective payload, and for many autistic learners it has a principled basis. Influential accounts characterise autism partly in terms of how prediction operates, describing difficulty in extracting reliable regularities from a noisy environment, so that a predictable, structured setting reduces the burden of uncertainty (Sinha et al., 2014; Van de Cruys et al., 2014). On this reading, the insistence on sameness often reported in autism is itself an adaptive search for predictive success, and a musical structure that supplies dependable regularity works with that grain rather than against it. A repeating, anticipable framework therefore offers a measure of safety, which is no minor matter given that anxiety is markedly more common in autistic populations (van Steensel et al., 2011), and a learner who feels secure within a task is better placed to take the small risks that creative exploration requires. This is not a loose intuition. Creativity anxiety specifically predicts poorer creative performance and the avoidance of creative tasks (Daker et al., 2020, 2023), so a structure that lowers it removes a documented barrier; and creative self-efficacy, the belief that one can produce creative work, supports the agentic step from potential to action (Beghetto, 2006; Karwowski & Beghetto, 2019). A predictable, secure frame plausibly acts on both.

Two qualifications keep this account honest, and both are built into the model rather than noted and forgotten. The first concerns sensory processing. A structured musical setting is only regulating if the sound itself is tolerable, and a substantial proportion of autistic learners experience sensory over-responsivity, an aversive, sometimes overwhelming reaction to particular auditory features such as timbre, volume, or density of texture (Yuan et al., 2022). For such a learner, a dense contrapuntal texture or a piercing instrumental timbre is not a neutral backdrop but a potential trigger for distress, and the regulation the model depends upon would fail at the first, physiological step. The framework therefore treats sensory profile not as a detail but as a first-order moderator and, at its limit, a contraindication: the sonic material must be selected for and with the individual learner, textures kept sparse where necessary, volume and timbre controlled, and a learner’s aversive response treated as a signal to change the material or stop, not as non-compliance to be managed. This is specified among the moderators in Section 6 and among the conditions of harm in Section 8.

The second qualification concerns the heterogeneity of attention and arousal, and it is where the “neurodiverse” label is most misleading if left unpacked. The regulatory logic above fits a profile of hyper-arousal and uncertainty-intolerance well, but learners with attention-related differences frequently present the opposite pattern. On long-standing optimal-stimulation accounts, some attentional profiles, prominently in ADHD, involve chronic under-arousal, so that novelty and stimulation are actively sought because they raise arousal toward an efficient level (Zentall & Zentall, 1983; Sergeant, 2005). For such a learner, a fixed, highly repetitive bass risks not safety but boredom, and the intervention could fail by under-stimulating rather than by overwhelming. The framework accommodates this not by exception but by design, for two reasons. First, a generative-constraint activity is never mere repetition: the ground bass is fixed, but the whole point of the activity lies in the variable, novelty-supplying layer above it, where the learner generates, changes, and controls the material. The predictable substrate and the stimulating surface coexist in a single activity, and their balance is adjustable. Second, the parameters that govern that balance, tempo, harmonic pace, the frequency and size of permitted variations, the length of a session, are exactly the tuning knobs a profile-sensitive design requires: faster, more variable, more novelty-rich realisations for a learner who needs arousal; slower, sparser, more predictable ones for a learner who needs calm. Recent transdiagnostic work on attention supports treating these profiles on a continuum rather than as fixed types, noting that intense, interest-driven focus and its converse, distractibility, can appear across autism and ADHD alike (Murray et al., 2005; Dwyer et al., 2024). The design principle that follows is the same in every case: match the constraint, and the stimulation it permits, to the learner’s arousal profile rather than assume a single profile for all.

This is the point at which the two halves of the thesis begin to meet, subject to those two qualifications. The same structural feature that channels the creative search (Section 3.1) also supplies, for a suitably matched learner, the regulation and security that allow entry into that search at all. Structure, properly understood, is doing affective and attentional work before it does any creative work, but only when its sensory material and its level of stimulation have been fitted to the individual.

### 3.3. Reframing the Mechanism: From Neuromyth to Regulated Conditions

The paper should be explicit about what it does not claim. It does not claim that listening to Baroque music makes students more intelligent, more attentive, or faster to learn. Claims of that kind belong to a family of educational neuromyths: beliefs that are loosely anchored in genuine science but that outrun the evidence and can distort practice (Dekker et al., 2012). It is worth being precise about the target of this correction, because contemporary music-therapy science long ago moved past the notion that passive listening confers cognitive benefit and now centres on active, participatory engagement. The neuromyth is addressed here not because it survives in that literature but because it survives, demonstrably, among educators and in popular practice, where it continues to be offered as the reason for playing “classical” music at learners; documenting its prevalence among teachers is precisely why studies of neuromyths were undertaken (Dekker et al., 2012). The aim is therefore not to refute a position the research community already rejects, but to clear away a folk rationale that still shapes classroom decisions and to put a defensible mechanism in its place. The framework’s positive commitment, developed below, aligns it squarely with the active-participation turn rather than with passive exposure.

Even granting the point, it is worth stating briefly why the “Mozart” rationale fails, since practitioners who hold it rarely encounter the evidence. The largest meta-analysis of the phenomenon finds little support for any specific, performance-enhancing effect of the music itself (Pietschnig et al., 2010), and experimental work shows that what improvement does appear is an artefact of arousal and mood: when differences in arousal and mood are held constant, the effect disappears (Thompson et al., 2001). The lesson is not that music is irrelevant to performance, but that its route runs through emotional and arousal states rather than through any structural property of the score. Music induces emotion through several mechanisms, among them brain-stem responses to sound and the play of musical expectancy, rather than through a single cognitive route (Juslin & Västfjäll, 2008), which fits an account that locates music’s contribution in affect and arousal rather than in cognition.

That lesson is exactly what the present framework needs. If music acts on arousal and affect, then its proper role in this account is regulatory, not cognitive. This repositioning brings the argument into line with the more cautious and better-supported evidence on active music-making in autism. A Cochrane review concludes that music therapy is probably associated with global improvement and with gains in quality of life and in overall symptom severity, while finding no clear effect on social interaction or communication measured immediately after intervention (Geretsegger et al., 2022). The honest reading of this evidence is modest but real: music-based work supports regulation, engagement, and social-emotional reciprocity rather than delivering broad cognitive uplift. A complementary review is more specific still, distinguishing educational from improvisational music therapy and reporting that the improvisational form is particularly associated with gains in social functioning (Mayer-Benarous et al., 2021), and a recent systematic review of creativity-focused interventions with autistic learners reaches compatible conclusions from the creativity side (Elisondo, 2025). These findings matter for the argument to come, because the musical practice at the centre of this paper is itself a form of structured improvisation, that is, active participation under constraint rather than passive listening.

Within this regulated frame, the appropriate yardstick for creative outcomes is not eminent achievement but the everyday, learning-embedded creativity that Kaufman and Beghetto (2009) term mini-c, together with the personally meaningful little-c that may follow. Holding the expectation at this level is not a concession; it is the conceptually correct target for creativity that is realised in the process of learning rather than in the historical record.

### 3.4. Stating the Model

**Figure 1:**
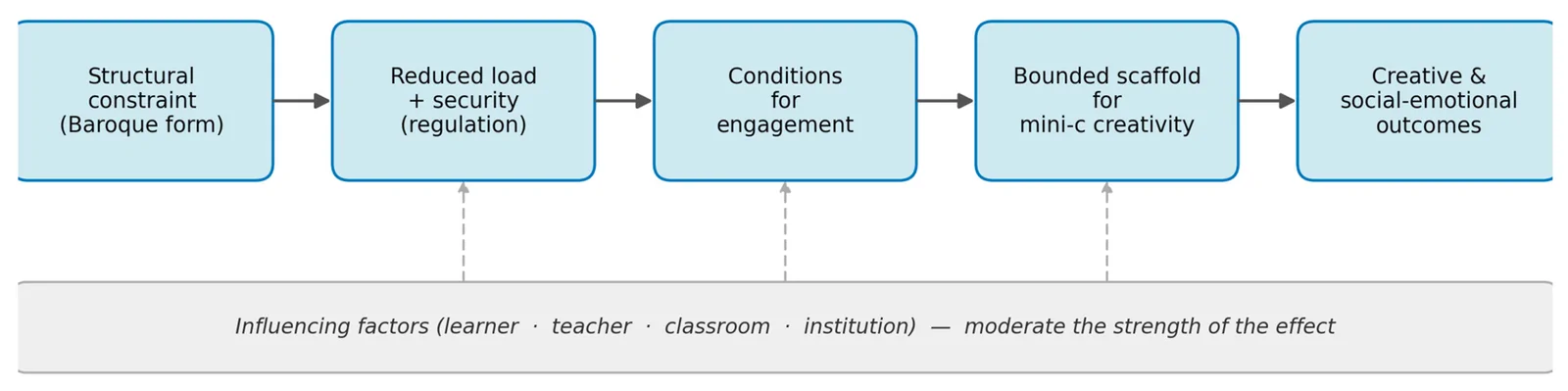
The generative-constraint model. A structural constraint supports regulation, which establishes the conditions for engagement, within which the structure scaffolds mini-c creativity; influencing factors moderate the strength of the chain. Solid arrows denote the sequential path of the model; dashed arrows denote the moderating influence of the influencing factors on the middle stages of the chain.

The generative-constraint thesis can now be stated as a single chain. A well-designed structural constraint channels the creative search and, for a suitably matched neurodiverse learner, lowers executive and sensory load while supplying a sense of security. That security and reduced load constitute the affective and attentional conditions for engagement. Within those conditions, the bounded structure functions not as a limit on expression but as a scaffold for it, and learning-embedded (mini-c) creativity becomes possible (Beghetto & Kaufman, 2007; Kaufman & Beghetto, 2009). Influencing factors at the level of the learner, the teacher, the classroom, and the institution do not generate this effect; they moderate how strongly it is realised, and are treated as such in Section 6. These stages, namely constraint, regulation, the conditions for engagement, the bounded scaffold, and mini-c production, together with the moderating role of the influencing factors, are exactly those set out visually in the model in Figure 1 (Section 3.4).

The chain also specifies its own failure modes, which is what allows it later to be tested rather than merely affirmed. If the sensory material is aversive, regulation fails at the first step; if the learner is chronically under-aroused and the realisation is kept monotonous, engagement never begins; and, most tellingly, if the constraint is enforced so rigidly that the variable layer collapses, the activity produces repetition without generation and the model’s central prediction, novel variation, goes unmet. These conditions of harm and failure are set out in Section 8, where they function as the disconfirming cases the framework must risk.

What remains is to show that a real musical tradition embodies this kind of constraint rather than merely tolerating it. The architecture of Baroque music fits this description with unusual precision. A repeating ground bass, or basso ostinato, holds a fixed lower layer constant while inviting endless melodic variation above it; theme-and-variation form prescribes a bounded set of transformations; and the historical practice of partimento and figured bass was, in its own time, a complete pedagogy of structured improvisation rather than an external scheme imposed on the music after the fact (Gjerdingen, 2007; Sanguinetti, 2012). The structure, in short, is not borrowed from elsewhere and laid over the music; it is the music’s own. Section 4 develops this correspondence in detail and is the analytic core of the paper.

## 4. Baroque Musical Architecture as Pedagogical Scaffold

Section 3 argued in the abstract that well-designed constraint scaffolds creativity, and that for a suitably matched neurodiverse learner the same structure supplies the regulation that makes engagement possible. This section makes the argument concrete. The claim is not that Baroque music happens to be compatible with such an approach, but that its characteristic forms are a paradigmatic case of generative constraint, and that in at least one of them the constraint was historically institutionalised as a method for teaching improvisation. The structure, on this account, is not imposed on the music from outside the classroom; it is the music’s own grammar.

Four features are considered: the ground bass, theme-and-variation form, the partimento and figured-bass tradition, and counterpoint. For each, the same two questions are asked. What creative operation does the feature scaffold, and what need of the neurodiverse learner does that scaffolding serve? The third subsection, on partimento, carries the most weight, because it is there that constraint and creative production are most clearly the same act. A concluding subsection asks why Baroque style in particular, rather than any other structured idiom, is a suitable vehicle.

### 4.1. Ground Bass (Basso Ostinato): A Fixed Foundation for Bounded Invention

A ground bass, or basso ostinato, is a short bass pattern repeated continuously while the music above it changes. The chaconne and passacaglia of the seventeenth and eighteenth centuries are built on this principle, and the widely familiar bass of Pachelbel’s Canon is a textbook instance. The lower layer is fixed and fully predictable; the upper layers are free to vary almost without limit.

The relevance to the generative-constraint thesis is immediate. The repeating bass is a constraint in Stokes’s (2006) sense: it does not restrict creative work arbitrarily but gives it a stable frame within which to operate. A learner improvising or composing over a ground does not face what composers experience as the “blank page,” by which is meant the unstructured, maximally open task, an unbounded set of possibilities with no starting constraint and therefore no obvious first move, which is cognitively and affectively demanding for exactly the reasons set out in Section 3.2. The foundation is instead given, anticipable, and unchanging, so the creative task is narrowed to a single manageable question, namely what happens above the bass. For a neurodiverse learner, the predictability of the lower layer does affective work before it does any creative work: a returning, knowable pattern offers the security discussed in Section 3.2, while the variable upper layer offers a low-stakes space for choice, and for a learner who needs stimulation rather than calm, that same upper layer is where novelty is introduced.

A clarification is needed here, because it bears directly on what makes the Baroque case more than a generic loop. A ground bass is not simply a pattern that recurs; it is a harmonic cycle with a built-in direction. Even the plainest Baroque ground, such as the descending tetrachord of the lament bass, sets up a functional motion from stability through tension to cadential resolution and back, so that each return of the pattern is also the renewal of an expectation of arrival (Meyer, 1956; Huron, 2006). This is precisely what a static ostinato, a rhythmic loop, or a lo-fi backing track need not possess: repetition alone provides regularity, but functional harmony provides regularity with a felt pull toward resolution. It is that pull, and not mere recurrence, that the learner’s melodic choices lean into or against, and it is what gives even a very simple activity a genuine musical, rather than merely textural, shape. Two further points keep this accessible. First, responding to functional harmony does not require producing or naming it: the tension and its resolution are heard and felt, so the perceptual demand is low even though the harmonic content is real, and the demanding work of realising the harmony is carried by the supplied frame, whether a recording, an app, or the teacher. Second, because the pull is predictable, it can be used gently, which is the basis of the flexibility mechanism developed in Section 5.5.

### 4.2. Theme and Variation: Transformation Within a Template

Theme-and-variation form states a musical idea and then transforms it through a series of variations that alter rhythm, dynamics, texture, mode, or instrumentation while preserving the identity of the original. The theme constrains what counts as a legitimate variation, and the recognised types of transformation constrain how the material may be changed.

In Stokes’s (2006) vocabulary, this approaches a novel goal paired with a defined set of operations for reaching it. The form is neither wholly open nor wholly closed: the learner is given a clear template and a bounded set of degrees of freedom along which to depart from it. This is pedagogically valuable precisely because it makes the creative move explicit and small. A student can be asked to change one parameter at a time, varying the rhythm while holding the pitches, or the dynamics while holding the rhythm, so that creative agency is exercised in steps whose scope the learner can see and control.

### 4.3. Partimento and Figured Bass: Structured Improvisation as Historical Pedagogy

The partimento and figured-bass tradition is the strongest case for the thesis, because in it the constraint is not a frame around improvisation but the very medium of improvisation. A figured bass is a written bass line annotated with numerals that specify the harmonies to be built above it; a partimento is an extended bass of this kind, used in the Italian conservatories of the eighteenth century as the basis for realisation at the keyboard. The student does not write an essay about harmony. The student sits at the instrument and improvises a full texture in real time from the figured line.

Two caveats must be stated at once, because the historical setting of partimento differs sharply from the one proposed here, and the difference could otherwise be mistaken for an equivocation. First, partimento was an elite conservatory method whose aim was professional mastery, the formation of what the Four C model would call Pro-c or Big-C musicians; it was not designed for, and is not here transplanted wholesale to, the special-education classroom. What the present argument borrows is not the eighteenth-century curriculum or its professional aims but a single pedagogical principle it exemplifies with unusual clarity, namely that improvisation can be taught, and creative fluency cultivated, by having a learner realise an explicit written pattern. That principle is scale-independent; the target here is mini-c, and the realisation is radically simplified. Second, the full figured-bass system is abstract and symbolically demanding, and Section 3.2’s distinction between extraneous and intrinsic load applies directly: for most neurodiverse learners the appropriate realisation of the principle is not numeral-annotated notation at all but its low-load descendants, a two-chord ground to improvise over, a single recurring pattern to fill in by ear, or a colour- or shape-coded scaffold on accessible software. For a minimally verbal or pre-notational learner, the “pattern” may be entirely non-symbolic, a heard and felt regularity realised by playing rather than by reading. The principle survives this simplification because it never depended on the notation; it depended on the presence of an explicit, repeatable frame within which a learner acts.

One inference from this simplification must, however, be blocked, because it marks the line between the present proposal and an ordinary accompaniment exercise. If the simplification were taken so far that nothing remained but a two-bar bass to sound freely over, the activity would no longer be partimento in any meaningful sense, and it would forfeit the very thing that distinguishes the tradition: partimento is a system of schemata, of voice-leading and harmonic implication, not a loop (Gjerdingen, 2007; Sanguinetti, 2012). What must survive simplification, then, is not a pattern of pitches but the schema’s harmonic logic. A lament bass still implies its cadential harmonisation; the Rule of the Octave still assigns each bass scale-degree its characteristic chord; a simplified two- or three-chord ground still moves from tonic through tension to resolution. When a learner shapes a melody over such a frame, the frame is doing functional-harmonic work whether or not the learner can name it, and the learner’s choices are meaningful precisely as responses to that harmonic motion. This is what separates the proposal from a backing-track exercise, and it is why the historical vocabulary is not decoration: the mechanism the tradition supplies, structured harmonic implication as a scaffold for invention, is exactly the mechanism the model needs.

With those caveats in place, the historical case can be stated for what it is. Sanguinetti (2012) characterises partimento as a teaching tool descended from basso continuo, designed to develop improvisation as a route to musical fluency, and Gjerdingen (2007) shows how training proceeded through a repertoire of conventional patterns, or schemata, that students internalised and recombined. Two points follow. First, the figured bass is a genuine constraint, since it fixes the harmonic skeleton and the voice-leading possibilities. Second, realising it is a genuinely creative act, performed under that constraint and in the moment. Constraint and creative production are therefore not sequential here, with structure first and freedom afterward; they are simultaneous, two descriptions of a single activity. This is what it means to say that the generative-constraint thesis is not applied to Baroque music from the outside: a central Baroque pedagogy already was a method for cultivating creative fluency by means of structure.

The pedagogical translation for the neurodiverse learner is correspondingly direct, provided the simplification just described is respected. A schema or figured pattern, in whatever simplified form suits the learner, provides an explicit scaffold; the learner fills it in, and in doing so gains both fluency and a sense of ownership over a result that is genuinely their own. The earlier evidence on improvisational music therapy is consistent with the value of this kind of work, in that Mayer-Benarous et al. (2021) associate the improvisational form specifically with gains in social functioning, the domain in which structured musical interaction is most plausibly beneficial.

### 4.4. Counterpoint: Rule-Bound Creativity

Counterpoint completes the picture more briefly. Writing two or more independent melodic lines that combine according to established rules of voice-leading is a densely constrained task, yet the space of acceptable solutions remains large. The rules do not dictate the music; they define the problem within which the music is found. For a learner who is comfortable with explicit systems, this kind of rule-bound problem-solving can be a congenial route into creative work, though its demands, both intrinsic-cognitive and, in dense textures, sensory, make it better suited to a later stage than the ground bass or the single-parameter variation, and unsuitable for learners with marked sensory over-responsivity to layered sound.

### 4.5. A Mapping of Affordances to Operations and Needs

Table 1 summarises the correspondence. Each musical feature is paired with the creative operation it scaffolds and with the learner need that scaffolding serves.

Together, these features show that the generative-constraint thesis has a detailed musical instantiation rather than a merely metaphorical one.

### 4.6. Why Baroque? A Comparison with Other Structured Idioms

Baroque music is not the only structured idiom, and the argument does not require it to be. It would be a weakness in the thesis if Baroque style were merely being contrasted with a vaguely defined, less-constrained alternative, so it is worth asking directly whether other idioms would serve as well, and what, if anything, is distinctive about the Baroque case. Several alternatives are genuine candidates. Later eighteenth-century “classical” style shares the theme-and-variation form and the fixed accompanimental patterns, such as the Alberti bass, that supply predictability. Nineteenth-century Romantic music is expressively powerful but tends to foreground rubato, dynamic surge, and harmonic surprise, features that work against the steady predictability the regulation stage depends on, and that may be poorly suited to a learner with sensory over-responsivity. Serialism is in one sense maximally constrained, but its constraints are abstract and its surface deliberately unpredictable, so it supplies rule-boundedness without the anticipable regularity that does the affective work here.

Two contrasts sharpen what is distinctive, and they turn on a single property: functional-harmonic directionality, the built-in motion from stability through tension to resolution described in Section 4.1. The first contrast is with approaches that supply predictable repetition without that directionality. The classic Orff-Schulwerk approach, for instance, builds improvisation on pentatonic and modal ostinati chosen precisely because they contain no dissonant tendency tones and so impose no obligation to resolve; this is pedagogically valuable and deliberately safe, but it removes the very ingredient the present mechanism relies on, the gentle pull toward an arrival that a learner’s choices can engage. Static loops, lo-fi backing tracks, and simple pop vamps share this limitation in varying degrees: they provide security through recurrence but little or no directional tension, and so they support comfortable participation more readily than the bounded, goal-directed invention the model is about. The second contrast is with jazz, which does possess functional-harmonic directionality in full measure, and here the honest conclusion is that jazz shares the core affordance. What separates the Baroque case is not the presence of the mechanism but its accessibility: jazz harmony is typically dense and rapidly changing, rich in extended and altered chords and idiomatic substitutions, and its improvisational culture presupposes a fluency that raises the entry cost for exactly the learners in view, whereas a Baroque ground reduces the same directional harmony to its simplest, slowest, most repeatable form. Baroque structure is therefore best understood as the clearest and lowest-threshold exemplar of directional functional harmony as a scaffold, flanked on one side by idioms that are safe but non-directional and on the other by idioms that are directional but demanding.

This also answers a fair question about contribution: if structured musical improvisation is already used in special education and music therapy, why frame it through Baroque music at all? The contribution is not the use of structured music, which is indeed established, but a specific mechanistic account of why and when it should work, namely that a predictable, directional harmonic frame regulates arousal and load and thereby scaffolds mini-c creativity, together with the moderator map that says for whom it should help, fail, or harm, and a measurement approach that distinguishes creative variation from mere repetition. Existing approaches such as Orff or improvisational music therapy are compatible with this account; what the model adds is an explanation of the active ingredient and a set of predictions that make the explanation testable, and, in the flexibility mechanism of Section 5.5, a principled reason to prefer a directional idiom when the therapeutic goal is to expand rather than merely to soothe. The choice of Baroque is thus a claim about clarity and historical warrant, not about exclusivity: the same mechanism should operate through any idiom that combines a predictable, directional harmonic substrate with an explicit, gradable pattern for improvisation, chosen to suit the learner’s sensory profile and, not least, their musical interest.

With the analysis in place, what remains is to turn it into a usable account of practice: how these affordances are arranged into activities, what conditions allow them to work, and how the resulting creativity might be recognised and assessed. Section 5 sets out that conceptual framework.

## 5. From Mechanism to Practice: A Conceptual Framework

The preceding sections supply the two halves of an argument: a general thesis that constraint scaffolds creativity under conditions of regulation (Section 3), and a demonstration that Baroque forms instantiate exactly this kind of constraint (Section 4). This section joins them into a single model and shows how the model translates into classroom practice. The aim is to make explicit what an earlier version of this work left implicit, and in particular to ground each practical strategy in a mechanism rather than in the discredited assumption that the music itself improves cognition.

### 5.1. The Generative-Constraint Model

The generative-constraint model is set out in Figure 1. It runs as a sequence. A structural constraint, supplied here by a Baroque musical form, reduces extraneous executive and sensory load and offers a predictable, secure frame. That reduction and security constitute the affective and attentional conditions for engagement. Within those conditions, the same structure now acts as a scaffold for creative production rather than as a limit on it, and the creativity it supports is of the everyday, learning-embedded kind that Kaufman and Beghetto (2009) call mini-c. The proximal results are creative and social-emotional rather than narrowly cognitive, consistent with the evidence reviewed in Section 3.3.

Two features of the model deserve emphasis. First, the model places regulation upstream of creativity rather than treating it as a side benefit, locating it deliberately between the constraint and the creative act. This is where the present account aligns with Amabile’s (1996) position that the environment is a component of the creative process and not merely its backdrop. In the vocabulary of creative self-beliefs, the regulation stage is where creativity anxiety is lowered and creative self-efficacy is supported, the agentic basis for moving from creative potential to creative action (Beghetto, 2006; Daker et al., 2020; Karwowski & Beghetto, 2019). Second, the familiar influencing factors discussed in the creativity-in-education literature, namely characteristics of the learner, the teacher, the classroom, and the institution, do not generate the effect. They moderate its strength, which is why they appear in Figure 1 as inputs to the middle of the chain rather than as its origin. Section 6 takes them up in detail.

### 5.2. Translating the Model into Practice

The strategies below are organised by the stage of the model they principally serve. None depends on the claim that exposure to Baroque music raises intelligence; each instead uses a specific musical affordance from Table 1 to do regulatory or scaffolding work. Table 2 maps the strategies onto the model.

*Structured listening for regulation*. Using a stable, moderately paced Baroque piece during transitions or settling periods targets the regulation stage directly. The rationale is arousal and mood management (Thompson et al., 2001), not cognitive enhancement, and the activity is framed accordingly as preparation for engagement rather than as a learning intervention in its own right. The choice of piece is made with the individual learner’s sensory tolerance in mind.

*Ground-bass improvisation.* Improvising a melody over a fixed repeating bass operationalises the scaffolding stage using the affordance analysed in Section 4.1. The constant lower layer supplies security, and the variable upper layer supplies a bounded space for creative choice, and, where a learner needs arousal rather than calm, the site at which novelty is introduced.

*Thematic variation.* Asking a learner to alter one parameter of a given theme at a time uses the theme-and-variation affordance of Section 4.2 to make the creative act explicit, small, and controllable.

*Multi-sensory integration.* Pairing listening with drawing, movement, or storytelling supports engagement and regulation across modalities, which can help learners whose sensory profiles make a single-channel task either under- or over-stimulating.

*Group ensemble and accessible technology.* Simple ensemble playing, or composition with accessible software, recruits the social dimension in which structured musical interaction is most plausibly beneficial (Mayer-Benarous et al., 2021), and extends the scaffolding logic to collaborative work. Accessible technology also matters for a reason taken up in Section 6.2: software that supplies the harmonic frame allows the scaffolding logic to operate even where the teacher is not a trained musician.

### 5.3. Application Principles

Three principles govern the use of these strategies and follow directly from the model. First, brief and regularly scheduled sessions are preferable to occasional long ones, because the regulatory benefit depends on predictability. Second, visual supports and clear routines reinforce the predictability that the model treats as causally central. Third, because the target outcome is mini-c creativity realised in process, evaluation should attend to the process rather than to a finished product; the form this evaluation takes is developed in Section 7. These principles are deliberately modest, and they make no claim beyond what the mechanism in Figure 1 supports.

### 5.4. The Scope of the Model: Purpose, Age, Population, and the Domain Question

Three questions about scope should be answered before the influencing factors are specified, because they bound what the model claims. The first concerns purpose. The primary aim of a generative-constraint activity is creative engagement together with the social-emotional regulation that makes it possible; the acquisition of specifically musical knowledge is a welcome by-product but not the point, and the framework should not be read as a music-curriculum proposal in disguise. Music is the vehicle because its architecture supplies unusually clean constraints, not because musical skill is the target outcome.

The second concerns age and generality. The mechanism, constraint reducing load and supplying regulation so that bounded creative choice becomes possible, is not age-specific, but its realisation is: the entry-level activities suit younger or less experienced learners, while richer realisations presuppose more, so the model is best understood as spanning the primary-to-secondary range with complexity titrated to the individual rather than to the year group, nor is the mechanism specific to neurodiverse learners. Constraint benefits creative work quite generally (Stokes, 2006), and the model would predict some benefit for typically developing learners too. The claim is one of degree, not of kind: the regulatory half of the model does its greatest work where load and arousal are most in need of management, which is disproportionately, though not exclusively, among neurodiverse learners. The framework is thus a special case, sharpened for a population where its central variables are most salient, of a more general truth about creativity and constraint.

The third question is more theoretical and was rightly pressed in review. There is a well-known tension in the creativity literature between treating creativity as domain-general and treating it as domain-specific (Baer, 1998), and mini-c sits awkwardly across it: mini-c is often described as a domain-general, personal-interpretive phenomenon in which detailed domain knowledge matters little, yet the Baroque vehicle proposed here is conspicuously domain-specific, both in the knowledge it presupposes and in the means of expression it offers. The apparent paradox dissolves once the two are assigned to their proper levels. The mechanism, constraint lowering load and enabling bounded generation, is domain-general; it is claimed to operate wherever a suitable constraint is supplied. The vehicle, a particular musical form, is domain-specific, and it is one vehicle among the several canvassed in Section 4.6. Mini-c itself was never domain-free in the strong sense: it was defined as the novel and personally meaningful interpretation that arises within the learning of some particular content (Beghetto & Kaufman, 2007), so a domain-specific medium is not in competition with mini-c but is exactly the kind of setting in which mini-c was said to occur. What is domain-general is the learner’s creative-interpretive act; what is domain-specific is the material through which it is exercised. This also makes plain an assumption that should not be left tacit, namely that the medium appeals to the learner. Where a given learner’s interests lie elsewhere, the same mechanism can in principle be carried by a different, better-fitting domain, and learner interest is accordingly treated as a moderator in Section 6.1 rather than assumed.

With the model, its practical translation, and its scope in place, the influencing factors named throughout can be treated systematically. Section 6 specifies them as moderators of the chain shown in Figure 1.

### 5.5. Using Harmonic Motion to Support Flexibility, Not Sameness

A distinct objection deserves a distinct answer: if the intervention fixes a low, repeating bass, does it not risk feeding the very insistence on sameness that characterises many autistic learners, entrenching rigidity rather than opening it? The concern is legitimate, and the model must do more than detect the danger after the fact; it must contain a positive mechanism that works against it. Functional-harmonic directionality supplies one. Because the harmonic frame is predictable, small departures from it are also predictable, and predictable novelty is tolerable novelty. The security of the returning ground can therefore be used as a platform from which flexibility is invited in graded, non-threatening steps that draw on Baroque music’s own characteristic devices. A prepared suspension introduces a moment of dissonance that the learner hears resolve, modelling tension and release within a safe frame. A brief tonicization or gentle modulation moves the ground to a neighbouring key and back, so that sameness is interrupted by a small, coherent excursion rather than by an arbitrary surprise. An added imitative voice, in the manner of simple counterpoint, invites a call-and-response that turns solitary repetition into a shared, turn-taking exchange. Each of these is a controlled, bounded destabilisation: it uses the frame’s predictability to make change feel safe, it is titrated to the individual, and it is introduced only once a secure baseline is established and withdrawn if it produces distress. This reframes the role of structure in the model. The fixed ground is not the therapeutic end in itself, which would indeed risk reinforcing rigidity; it is the secure base from which directional harmony coaxes flexibility. Used this way, the characteristic resources of Baroque music, its suspensions, its cadential drive, its modulations, and its contrapuntal interplay, are not incidental ornaments but the active means by which the approach works with, rather than against, the goal of expanding a learner’s range. Section 8 states the corresponding prediction and the stopping rules that protect a learner for whom it does not work.

## 6. Influencing Factors

The model in Figure 1 distinguishes the mechanism that produces creative engagement from the factors that modulate how strongly it operates. This section specifies those factors. The distinction is not merely organisational. Treating learner, teacher, classroom, and institutional characteristics as moderators rather than as causes keeps the explanatory weight on the constraint-and-regulation chain and prevents the familiar slide into claiming that any single resource or trait produces creativity. It also matches the framing of influencing factors as conditions that shape outcomes rather than guarantee them.

### 6.1. Learner-Level Factors

Characteristics of the individual learner condition the strength of the effect at every stage of the model. Age and developmental level bear on which musical affordances are accessible: a single-parameter thematic variation may suit a younger or less experienced learner, while figured-bass realisation presupposes more. A learner’s profile, meaning the specific configuration of attentional, sensory, and communicative strengths and differences, shapes how much regulatory benefit a given structure provides and how much scaffolding a creative task requires. Two dimensions of that profile are singled out here because the model gives them decisive weight. The first is the sensory profile: as Section 3.2 argued, sensory over-responsivity to particular auditory features can turn a regulating setting into an aversive one (Yuan et al., 2022), so timbre, texture, and volume must be selected for and with the learner, and an aversive response is treated as a contraindication rather than as non-compliance. The second is the arousal and attentional profile: a learner who is chronically under-aroused and novelty-seeking needs a faster, more variable, more stimulating realisation than a learner who is hyper-aroused and uncertainty-intolerant (Zentall & Zentall, 1983; Sergeant, 2005; Dwyer et al., 2024), so the same activity is tuned in opposite directions for the two. Prior musical experience raises the ceiling on what counts as a bounded but reachable creative move, and learner interest, whether the medium engages the learner at all, conditions whether the mechanism has any purchase, which is why Section 5.4 treats an unappealing medium as a reason to change the vehicle. Throughout, these are framed as moderators to be matched, not deficits to be corrected; the design task is to fit the constraint to the learner rather than to standardise the learner to the constraint. Intrinsic task motivation, which Amabile (1996) identifies as especially conducive to creative work, is both a learner characteristic and a target of good design. Two affective-motivational characteristics deserve particular mention as moderators, since the model assigns them a central role. A learner’s creative self-efficacy and creative mindset condition how readily the secure frame translates into engagement (Beghetto, 2006; Karwowski, 2014), while a learner’s level of creativity anxiety conditions how much regulatory work the structure must do (Daker et al., 2020). These are not fixed traits but themselves targets that sustained, repeated activity may shift.

### 6.2. Teacher-Level Factors

The teacher is the most consequential moderator, and the demand the approach appears to place on teachers deserves a direct and candid answer, because taken at face value it looks unrealistic. On the strongest reading, the teacher must command both real musical craft, enough to set up and guide a ground bass, a thematic variation, or a simple realisation, and skilled special-education practice, enough to regulate the affective and sensory environment in which that work occurs. Practitioners who embody both, the “dual expert,” are genuinely rare, and a framework whose preconditions almost never obtain in real settings would be of little practical use however elegant its theory. The objection is well taken, and the model’s answer is that this maximal reading is not in fact required; the dual competence is a property the delivery system must possess, not a property each individual teacher must possess.

Four routes make the approach ecologically realistic without positing rare individuals. First, and most obviously, the two competencies can be split across two people through co-teaching or consultation: a visiting or specialist music educator supplies the musical craft while the special educator, who knows the learners, manages regulation, a division of labour already common in inclusive settings. Second, the musical demand at the entry level is modest and is frequently misjudged by reading it against the partimento end of the spectrum; guiding a learner to vary a rhythm over a pre-recorded two-bar bass requires far less musical training than realising a figured bass, and most of the strategies in Section 5.2 sit at this low-demand end. Partimento proper is the advanced, optional ceiling of the approach, not its floor, and no ordinary special educator is asked to “operate partimento.” Third, accessible technology substitutes for musical expertise at precisely the point where it is scarcest: software that supplies the harmonic frame, loops the ground, or constrains the available notes lets a non-musician teacher offer a sound scaffold, so that the teacher’s contribution can concentrate on regulation and encouragement. Fourth, the specific, bounded skills the approach does require of the classroom teacher are trainable through short professional-development modules rather than presupposing a conservatory background. The realistic precondition of the model is therefore an appropriately resourced delivery system, one teacher with training and technology, or two teachers with complementary expertise, not a population of unicorns.

Beyond the question of competence, the benefit depends on fidelity to the scaffolding logic, and this is a distinct and trainable skill. A teacher who, with good intentions, removes the constraint in the name of free expression dismantles the very structure that does the regulatory and creative work; a teacher who enforces the constraint so rigidly that no genuine choice remains eliminates the creative act itself and, as Section 8 notes, risks reinforcing repetition rather than generation. The teacher’s task is to hold the constraint while opening the bounded space within it, and this balance, rather than any rare fusion of two careers, is the genuine skill the approach asks teachers to acquire.

### 6.3. Classroom and Institutional Factors

At the level of the classroom, the physical and sensory environment either supports or undermines the regulation stage of the model. Predictable routines, manageable sensory load, and reliable access to appropriate instruments or accessible technology strengthen the chain, and their absence weakens it. Large-scale evidence is consistent with the weight placed on the classroom: in PISA 2022, students who reported that their teachers valued and encouraged their creativity scored somewhat higher in creative thinking, even after accounting for student and school characteristics (OECD, 2024). At the institutional level, curricular flexibility determines whether brief, regular creative sessions can be embedded in the timetable, and resource support determines whether the necessary training, technology, and specialist time identified in Section 6.2 are actually available. These institutional factors are easily overlooked because they are not visible in the moment of teaching, yet they set the outer limits on what the classroom and the teacher can achieve. The overall claim of this section is therefore cumulative: the effect described in Section 5 is realised to the degree that learner, teacher, classroom, and institution are aligned, and is attenuated wherever they are not.

## 7. Outcomes and Their Measurement

If the influencing factors set the conditions, the question remains what the approach is expected to achieve and how that achievement could be recognised. This section addresses both, under an explicit constraint of its own. As a conceptual contribution, the paper proposes a model and does not report data, so its claims about outcomes are claims about what should be looked for and how, not demonstrations that the looking has succeeded. Keeping this distinction visible is essential to the paper’s credibility, and it corrects a tendency, present in earlier and less cautious treatments, to assert measured benefits that the evidence base does not support.

### 7.1. What Counts as an Outcome

The model predicts outcomes of two kinds, and pointedly not a third. It predicts creative outcomes understood at the level of mini-c (Beghetto & Kaufman, 2007; Kaufman & Beghetto, 2009): a learner’s generation of musical ideas that are novel and meaningful to them within the bounded space of the task. It predicts social-emotional outcomes, including reduced anxiety, greater willingness to engage, and, in collaborative settings, improved reciprocity, which is consistent with the modest but real findings of the music-therapy evidence (Geretsegger et al., 2022). It does not predict broad cognitive uplift, such as gains in general intelligence, memory, or attention that transfer beyond the activity. That third category is precisely the neuromyth rejected in Section 3.3, and the present framework neither requires nor claims it.

### 7.2. Measuring mini-c Creativity

Measuring creativity at the level of mini-c is genuinely difficult, because mini-c is defined by personal meaning and is realised in process rather than deposited in a finished, comparable product. The difficulty is real, but it does not leave the construct beyond disciplined observation, and the design must be built so that the central dependent variable is not simply the intervening teacher’s impression. Three approaches, used together, are appropriate. First, the dimensions established in the divergent-thinking tradition, namely fluency, flexibility, originality, and elaboration (Torrance, 1966), can be adapted to musical tasks, with originality assessed against the learner’s own repertoire and context rather than against a population norm, in keeping with the appropriateness criterion of the standard definition (Plucker et al., 2004) and with the learner-referenced reading of appropriateness defended in Section 2.1. Several of these dimensions yield countable, relatively objective indicators, the number of distinct variations a learner produces over a fixed ground, the number of parameters they manipulate, the emergence of a combination not previously modelled, which can be coded from a record of the session without recourse to the observer’s inference about inner states. Second, process-based methods, including structured observation and portfolios that capture successive attempts, are better suited than single end-of-task products to a construct that lives in the doing. Third, because mini-c is partly an internal event, the learner’s own account of what they intended and found meaningful is itself evidence rather than an optional supplement. This last source, however, presupposes a degree of verbal report that not every learner can give. A substantial share of autistic children remain minimally verbal into the school years (Tager-Flusberg & Kasari, 2013), and for them self-report cannot carry evidential weight; the strategy must then rest on the first two sources, drawing on assessment approaches developed specifically for minimally verbal learners, including structured observation of engagement and behaviour and the use of non-verbal and caregiver-informed measures (Kasari et al., 2013), so that the verbal account is replaced rather than the activity judged closed to these learners.

A specific hazard must be confronted before any of these indicators can be trusted, because it goes to the heart of the construct in this population. If creative production is operationalised simply as variation produced over a repeating musical frame, then for an autistic learner some of that variation could be repetitive or self-stimulatory behaviour, stimming or motor stereotypy, rather than creative choice, and a naive count of variations would confound the two (Leekam et al., 2011). The distinction matters both scientifically, because the model claims to produce creativity and not merely to elicit existing behaviour, and ethically, because stimming is a valued form of self-regulation that should be neither pathologised nor mistaken for something it is not (Kapp et al., 2019). The response is to define the dependent variable not as variation as such but as structure-responsive variation, and to give raters explicit, low-inference criteria that separate it from stereotypy. Four are used here. The first is contingency on the frame: creative production changes when the musical structure changes, whereas stereotyped behaviour tends to continue unaltered regardless of harmonic or rhythmic context, so probing the learner with an altered frame, a modulation, a changed harmony, a pause, and observing whether the output tracks the change, is diagnostic. The second is structural alignment: creative choices bear an audible relation to the harmonic motion, landing on or leaning against points of tension and resolution, whereas stereotypy is typically indifferent to them. The third is novelty relative to the learner’s own habitual repertoire: a move that departs from the learner’s established, characteristic self-stimulatory patterns is more plausibly generative than one that reproduces them. The fourth is communicative directedness: shared attention, turn-taking, and orientation to a partner mark the act as socially embedded creation rather than self-directed regulation. These criteria are applied by independent raters informed by a functional–behavioural understanding of the individual learner’s baseline repetitive behaviours, so that the coding rests on the relation between behaviour and structure rather than on the mere occurrence of repetition. This does not make the measurement effortless, but it makes the crucial distinction observable, and it converts a genuine threat to validity into an explicit, testable part of the design.

Proxy and observational measures of this kind carry their own hazard, and because the model’s credibility depends on the answer, it must be met squarely rather than merely acknowledged. An observer infers an internal, personally meaningful experience from outward behaviour and may read it through their own expectations; where the observer is also the teacher who delivered the activity and hopes to see it succeed, that expectancy can inflate the scores, and the danger is compounded by the fact, discussed in Section 8.2, that the delivering teacher usually cannot be blinded. The response is to move the central measurement off the delivering teacher’s subjective judgement wherever possible: to privilege the countable, low-inference indicators described above; to have creative output coded by independent raters who took no part in delivery and, where feasible, who score from video records blind to the study phase; and to report inter-observer agreement so that the reliability of the coding is itself on view. Proxy bias cannot be eliminated where an internal state is at issue, but a dependent variable built from independently coded, low-inference behavioural indicators is a genuine measurement rather than a restatement of the teacher’s hopes, and it is on that basis, developed further in Section 8, that the model’s claim to be testable rests. The aim of measurement here is formative and descriptive, oriented to understanding and supporting the individual learner, not to summative ranking.

### 7.3. The Limits of Claims

Two limits follow and should be stated plainly. The first is inferential: an internal, personally meaningful creative event cannot be observed directly, so any measure is a partial proxy whose limitations must be acknowledged rather than glossed. The second is evidential: nothing in this paper establishes the magnitude, durability, or generality of the outcomes it describes, and language implying that the approach “significantly enhances” creativity would misrepresent a conceptual proposal as an empirical result. What the paper offers instead is a model precise enough to be tested, together with an account of what evidence would bear on it. Section 8 sets out how that test might be designed.

## 8. A Research Agenda

A conceptual paper earns its place not by demonstrating its claims but by making them precise enough to be tested and by indicating how a test would proceed. This section discharges that obligation. It derives testable predictions from the model of Section 5, including predictions about the conditions under which the approach should fail or do harm, identifies the class of research design suited to evaluating them in this population, and sketches a path from individual studies to cumulative evidence. The absence of data here is not a gap to be apologised for; supplying the design under which data could be gathered is the proper work of a framework of this kind.

### 8.1. Testable Predictions

The model in Figure 1 yields several predictions specific enough to be wrong. The first concerns the basic effect: relative to an open, weakly structured musical activity, a bounded generative-constraint activity should produce greater task engagement and a higher rate of mini-c production (Kaufman & Beghetto, 2009) for a suitably matched neurodiverse learner. The second concerns the mechanism: the model holds that structure acts on creative engagement by way of regulation, which predicts that indices of regulation and reduced load, whether physiological, such as heart-rate or electrodermal measures of arousal, or behavioural, such as time on task, latency to engage, and observable signs of distress, should change before, and statistically account for, changes in creative engagement, rather than the two moving independently. The third concerns moderation: the strength of the effect should vary systematically with the factors of Section 6, and in particular should be larger where teacher fidelity to the scaffolding logic is higher, where the sonic material has been matched to the learner’s sensory profile, and where the level of stimulation has been matched to the learner’s arousal profile. A fourth prediction follows from the music-therapy evidence, in that an improvisational, partimento-like format should yield larger social-functioning gains than a listening-only format (Mayer-Benarous et al., 2021).

A fifth class of prediction concerns harm and failure, and the model is stronger for stating it, because a framework that can specify when an intervention should backfire is more falsifiable, not less. Three conditions of harm follow directly from the mechanism. Where the auditory material exceeds a learner’s sensory tolerance, the model predicts not a smaller benefit but a reversal, distress and disengagement, appearing at the regulation stage before any creative stage is reached; this is why sensory profile functions as a contraindication and not merely as a dial. Where a learner is chronically under-aroused and the realisation is held monotonous, the model predicts boredom and withdrawal rather than engagement, an under-stimulation failure distinct from the sensory one. And where the constraint is enforced so rigidly that the variable layer collapses, the model predicts the outcome the reviewer of an earlier version rightly worried about: repetition without generation, which for a learner already inclined to sameness could entrench rigidity rather than open it. The framework’s defence against that last danger is not a promise that it cannot happen but a criterion for detecting it, namely that the presence of genuine, novel variation, and not mere repetition, is the very thing the measurement of Section 7.2 is designed to register. An activity that produces only repetition has, by the model’s own account, failed, and the design is built to show this. Each of these predictions admits a result that would count against the model, which is what makes the model more than a redescription.

A sixth prediction concerns the flexibility mechanism of Section 5.5 and is the ethical counterpart of the harm prediction. If the guided introduction of harmonic novelty, resolving suspensions, brief modulations, and imitative exchange, works as claimed, then a condition that adds this graded destabilisation to the secure ground should reduce, not increase, indices of rigidity and perseveration relative to a condition that holds the ground static, and should do so without a rise in signs of distress. The model thus predicts that the correct use of structure moves a learner toward flexibility; should the opposite occur, so that static repetition proves as good or better while destabilisation raises distress, the mechanism as stated would be disconfirmed. This prediction carries a built-in safeguard. Because rigidity and distress are monitored as outcomes in their own right, the design specifies stopping rules, so that an individual for whom the approach entrenches sameness or provokes distress is withdrawn from it rather than continued in the name of the protocol. Detecting harm is therefore not left to post hoc interpretation; it is an outcome the design is obligated to watch for and to act on. So specified, the concern that a fixed harmonic frame might reinforce pathological sameness is not merely conceded but converted into a monitored, falsifiable prediction with a protective response attached, which is what a clinically responsible model owes it.

### 8.2. Appropriate Designs: Single-Case Experimental Designs

The design best suited to evaluating these predictions in special education is not the randomised controlled trial but the single-case experimental design (SCED). The reasons are principled rather than merely pragmatic. The relevant population is small and highly heterogeneous, so group averages can obscure precisely the individual variation that the moderation predictions are about; and the construct of interest, mini-c, is realised in the individual learner over repeated occasions rather than in a between-groups contrast. Single-case methods meet these conditions directly: each participant serves as their own control, the dependent variable is measured repeatedly across baseline and intervention phases, and experimental control is established by replicating the effect within and across participants (Horner et al., 2005).

A multiple-baseline design across participants, behaviours, or settings is a natural fit, since it can show that creative engagement rises when, and only when, the generative-constraint activity is introduced. It is also the appropriate response to a feature of this subject matter: because the target is learning, its effects are not expected to reverse once the activity is withdrawn, which makes withdrawal and reversal designs ill-suited and means that the carry-over of skills across phases, rather than confounding the design, is anticipated and accommodated by staggering the activity’s introduction across participants or settings. There is an ethical dimension to the same choice: withdrawing a support that appears to be helping a learner, solely to demonstrate experimental control, is difficult to justify in a special-education setting, which is a further reason to prefer multiple-baseline logic over withdrawal or reversal. This repeated, individualised measurement also aligns with the process-based and formative assessment argued for in Section 7. The standards for conducting and appraising such work are well established (Horner et al., 2005; Kratochwill et al., 2010) and include criteria for the description of participants and settings, the definition of dependent and independent variables, baseline conditions, internal and external validity, and social validity.

One threat to internal validity deserves particular attention here, because it was pressed in review and because the model’s scientific standing turns on it: when the teacher who delivers the activity is also the person who rates the learner’s creative output, their expectations can shape the scores, and that delivering teacher usually cannot be blinded. This is a real threat, but it is a threat the single-case tradition has established means to control, and the response has two parts. The first is measurement design, carried over from Section 7.2: the dependent variable is built as far as possible from countable, low-inference indicators of generativity, such as the number of distinct variations produced, rather than from a global impression of creativity, so that less is left to inference in the first place. The second is independent, blinded coding: creative output is coded by raters who took no part in delivery, wherever possible, from video records scored blind to the study phase, with inter-observer agreement reported. Blinding the scorer, even when the deliverer cannot be blinded, breaks the link between the deliverer’s expectancy and the recorded outcome, which is the specific mechanism of the expectancy effect. A dependent variable so constructed is not a subjective inference dressed as data; it is an independently verifiable behavioural record, and it is on this basis that the model’s predictions can be genuinely confirmed or disconfirmed. Designing to these standards from the outset is what would allow individual studies to contribute to a credible evidence base rather than remaining isolated demonstrations.

### 8.3. Toward Cumulative Evidence

A recurring worry about single-case work is that its very individualisation dooms it to a heap of anecdotes from which nothing general can be drawn, and if that were so, a framework resting on SCED could never become an educational theory. The worry misreads how the tradition generalises. Single-case findings become general not through any one decisive study but through systematic replication: in this tradition a practice is regarded as evidence-based only once an effect has been reproduced across multiple studies, research teams, and participants (Horner et al., 2005). What generalises, moreover, is not the claim that a fixed “Baroque constraint” affects every child identically, which the heterogeneity of learners would indeed refute, but the claim that the mechanism, constraint enabling regulation and so bounded generation, holds across learners whose specific parameters differ. The individual differences the objection emphasises are, on this model, not noise that swamps a law but the very content of the moderation predictions of Section 6: replication across sensory profiles, arousal profiles, ages, and levels of teacher fidelity converts those moderators from conjectures into a map of the conditions under which, and the learners for whom, the approach works best and worst. A programme of work planned with that threshold and that structure in view yields exactly the nomothetic product the objection supposes to be unreachable, an account of a general mechanism together with the boundary conditions on its expression. Larger-scale or mixed designs may become appropriate once the basic effect and its boundary conditions are established, but they are not the place to begin.

Throughout, the model remains falsifiable. Were structured constraints to leave engagement and mini-c production unchanged, or were its effects to bypass regulation entirely, or were the conditions of harm specified in Section 8.1 not to appear where the model says they must, the framework advanced here would be disconfirmed. It is in this openness to disconfirmation, as much as in its synthesis of existing work, that the contribution is intended to be scientific rather than merely persuasive.

## 9. Conclusions

This paper has argued that the value of Baroque music for creativity in special education has been misattributed. The benefit does not lie in any power of music to improve cognition, a claim that the evidence does not support. It lies instead in the way structured musical environments establish the conditions, principally regulation and security, under which everyday, learning-embedded creativity can emerge in neurodiverse learners, when the sensory material and the level of stimulation have been fitted to the individual. The thesis of generative constraint names this mechanism, and Baroque musical architecture, in the ground bass, in theme-and-variation form, and above all in the historical pedagogy of partimento and figured bass, supplies a strikingly precise embodiment of it. In partimento especially, constraint and creative production are not sequential but simultaneous, which is why a central Baroque pedagogy can be described, without strain, as a method for teaching creativity through structure, provided its elite origins are translated into radically simplified, learner-matched form.

Two commitments have shaped the argument and should outlast it. The first is honesty about claims: the paper offers a conceptual model and a research agenda, not evidence of effect, and it has been careful to promise no more than that, and to specify the conditions under which the approach should be expected to fail. The second is a strength-based stance toward neurodiversity, one that asks what conditions allow a learner’s creative capacity to be expressed rather than treating difference as a deficit to be managed, and that treats the real differences among neurodiverse learners as design parameters rather than as inconvenient exceptions. If the framework is right, the practical task for educators is neither to play the right music at students nor to remove all structure in the name of freedom, but to design constraints well: to hold a secure, predictable frame while opening, within it, a bounded space in which a learner’s own ideas can take shape. Whether the model is right is an empirical question, and the paper has tried to pose it precisely enough to be answered.

## Figures and Tables

**Table 1:** Baroque musical affordances mapped to the creative operations they scaffold and the learner needs they serve.

Musical Affordance	Creative Operation Scaffolded	Learner Need Served
Ground bass (basso ostinato)	Bounded invention over a fixed foundation	Predictable, anticipable base that lowers anxiety and cognitive load
Theme and variation	Systematic transformation of a fixed idea	Clear template with small, controllable degrees of freedom
Partimento/figured bass	Real-time improvisation from an explicit written pattern	Concrete scaffold yielding fluency and creative ownership
Counterpoint	Rule-bound combination of independent lines	Explicit system supporting stepwise creative problem-solving

**Table 2:** Practical strategies mapped onto the stage of the model they principally serve and the Baroque affordance each draws on.

Strategy	Model Stage Principally Served	Baroque Affordance Drawn on
Structured listening	Regulation	Stable, moderately paced listening
Ground-bass improvisation	Scaffolding → mini-c creativity	Ground bass (Section 4.1)
Thematic variation	Scaffolding → mini-c creativity	Theme and variation (Section 4.2)
Multi-sensory integration	Engagement (cross-modal)	Listening paired with other modalities
Group ensemble & technology	Engagement & social outcomes	Ensemble play; accessible composition

## Data Availability

No new data were created or analyzed in this study. Data sharing is not applicable to this article.
